# Biodistribution Assessment of a Novel ^68^Ga-Labeled Radiopharmaceutical in a Cancer Overexpressing CCK2R Mouse Model: Conventional and Radiomics Methods for Analysis

**DOI:** 10.3390/life14030409

**Published:** 2024-03-20

**Authors:** Anna Maria Pavone, Viviana Benfante, Paolo Giaccone, Alessandro Stefano, Filippo Torrisi, Vincenzo Russo, Davide Serafini, Selene Richiusa, Marco Pometti, Fabrizio Scopelliti, Massimo Ippolito, Antonino Giulio Giannone, Daniela Cabibi, Mattia Asti, Elisa Vettorato, Luca Morselli, Mario Merone, Marcello Lunardon, Alberto Andrighetto, Antonino Tuttolomondo, Francesco Paolo Cammarata, Marco Verona, Giovanni Marzaro, Francesca Mastrotto, Rosalba Parenti, Giorgio Russo, Albert Comelli

**Affiliations:** 1 Section of Physiology, Department of Biomedical and Biotechnological Sciences, University of Catania, 95123 Catania, Italy; ampavone@fondazionerimed.com (A.M.P.); r.vincenzosimone@gmail.com (V.R.); parenti@unict.it (R.P.); 2 Ri.MED Foundation, Via Bandiera 11, 90133 Palermo, Italy; vbenfante@fondazionerimed.com (V.B.); pgiaccone@fondazionerimed.com (P.G.); acomelli@fondazionerimed.com (A.C.); 3 Department of Health Promotion, Mother and Child Care, Internal Medicine and Medical Specialties, Molecular and Clinical Medicine, University of Palermo, 90127 Palermo, Italy; bruno.tuttolomondo@unipa.it; 4 Research Unit of Computer Systems and Bioinformatics, Department of Engineering, Università Campus Bio-Medico di Roma, Via Alvaro del Portillo 21, 00128 Rome, Italy; m.merone@unicampus.it; 5 Institute of Molecular Bioimaging and Physiology, National Research Council, IBFM-CNR, 90015 Cefalù, Italy; selene.richiusa@ibfm.cnr.it (S.R.); francesco.cammarata@ibfm.cnr.it (F.P.C.); giorgio-russo@cnr.it (G.R.); 6 Laboratori Nazionali del Sud, National Institute for Nuclear Physics, INFN-LNS, 95123 Catania, Italy; 7 Medicine and Surgery Department, University of Enna “Kore”, 94019 Enna, Italy; filippo.torrisi@unikore.it; 8 Legnaro National Laboratories, Italian Institute of Nuclear Physics, Viale Dell’Università 2, 35020 Padova, Italy; davide.serafini@lnl.infn.it (D.S.); luca.morselli@lnl.infn.it (L.M.); alberto.andrighetto@lnl.infn.it (A.A.); 9 Department of Physical Sciences, Earth and Environment, University of Siena, 53100 Siena, Italy; 10 Nuclear Medicine Department, Cannizzaro Hospital, 95126 Catania, Italy; marco.pometti@gmail.com (M.P.); fabrizioscopelliti@gmail.com (F.S.); ippolitomas@yahoo.it (M.I.); 11 Pathologic Anatomy Unit, Department of Health Promotion, Mother and Child Care, Internal Medicine and Medical Specialties, University of Palermo, 90127 Palermo, Italy; giulio.giannone@unipa.it (A.G.G.); cabibidaniela@virgilio.it (D.C.); 12 Radiopharmaceutical Chemistry Section, Nuclear Medicine Unit, AUSL-IRCCS di Reggio Emilia, Viale Risorgimento 80, 42122 Reggio Emilia, Italy; mattia.asti@ausl.re.it; 13 Department of Pharmaceutical Sciences, University of Padova, Via Marzolo 5, 35131 Padova, Italy; elisa.vettorato@unipd.it; 14 Department of Physics and Astronomy, University of Padova, Via Marzolo 8, 35131 Padova, Italy; marcello.lunardon@pd.infn.it; 15 Department of Pharmaceutical and Pharmacological Sciences, University of Padova, 35131 Padova, Italy; marco.verona@studenti.unipd.it (M.V.); giovanni.marzaro@unipd.it (G.M.); francesca.mastrotto@unipd.it (F.M.)

**Keywords:** ^68^Ga-labeled radiopharmaceutical, biodistribution, micro-PET/CT, mouse imaging, radiomics, nuclear medicine, histology, digital pathology

## Abstract

The aim of the present study consists of the evaluation of the biodistribution of a novel ^68^Ga-labeled radiopharmaceutical, [^68^Ga]Ga-NODAGA-Z360, injected into Balb/c nude mice through histopathological analysis on bioptic samples and radiomics analysis of positron emission tomography/computed tomography (PET/CT) images. The ^68^Ga-labeled radiopharmaceutical was designed to specifically bind to the cholecystokinin receptor (CCK2R). This receptor, naturally present in healthy tissues such as the stomach, is a biomarker for numerous tumors when overexpressed. In this experiment, Balb/c nude mice were xenografted with a human epidermoid carcinoma A431 cell line (A431 WT) and overexpressing CCK2R (A431 CCK2R+), while controls received a wild-type cell line. PET images were processed, segmented after atlas-based co-registration and, consequently, 112 radiomics features were extracted for each investigated organ / tissue. To confirm the histopathology at the tissue level and correlate it with the degree of PET uptake, the studies were supported by digital pathology. As a result of the analyses, the differences in radiomics features in different body districts confirmed the correct targeting of the radiopharmaceutical. In preclinical imaging, the methodology confirms the importance of a decision-support system based on artificial intelligence algorithms for the assessment of radiopharmaceutical biodistribution.

## 1. Introduction

Oncological researchers are seeking effective methods to overcome the technological limitations of current medicine by exploring alternatives to traditional therapies [1,2,3]. Nuclear medicine imaging is expanding rapidly, offering innovative imaging approaches for acquiring both molecular and anatomical information [4,5]. Over the past few decades, combined-modality imaging has played a crucial role in achieving this aim, providing various imaging techniques such as positron emission tomography (PET) [6], magnetic resonance imaging (MRI) [7,8], single-photon emission computed tomography (SPECT) [9] and computed tomography (CT) [10].

PET/CT or SPECT/CT represents the gold standard approach in many tumor assessments, enabling quantitative imaging investigations that provide both morphological and functional knowledge [11]. These techniques involve the use of specific molecules, known as radiopharmaceuticals, which consist of biological ligands labeled with unstable radionuclides. Depending on the radionuclide decay (α, β^+^ and β^−^, γ emissions and Auger electrons), radiopharmaceuticals can be employed for innovative diagnostic, therapeutic and theranostic applications [4,12]. The most commonly used radiopharmaceuticals in therapies exploit α, β- and Auger electron decays. α-decay radionuclides, such as actinium-225 (^225^Ac) and radium-223 (^223^Ra), release their energy over a short range, utilizing both direct and indirect anticancer effects through double-strand DNA breaks and the production of reactive oxygen species (ROS), respectively [13,14,15]. In contrast, β-emitting radionuclides are employed in radio-immuno targeting and targeted radiotherapy [16]. Therefore, α, β^−^ or Auger electron emitters find applications in therapeutic contexts, as opposed to β^+^ and γ emitters, including fluorine-18 (^18^F), technetium-99m (^99m^Tc) and gallium-68 (^68^Ga), which are commonly used in the diagnostic field [17,18].

F-18-fluorodeoxyglucose (^18^F-FDG) is a widely utilized radiopharmaceutical, approved by the Food and Drug Administration (FDA), and employed for detecting glucose metabolism in tumor physiology. It evaluates cancer glycolytic efficiency in various clinical fields, including neurology, cardiology and oncology [19]. Another β^+^-emitter is ^68^Ga, with a half-life of 68 min, employed in diagnostic molecular imaging [4]. Specifically, [^68^Ga]Ga-2,2′,2″,2‴-(1,4,7,10-Tetraazacyclododecane-1,4,7,10-tetrayl)tetraacetic acid (DOTA)-TOC enables the detection of somatostatin receptor (SSTR) expression in neuroendocrine tumors (NETs). This aids in predicting potential responses to radiopharmaceutical therapy, gaining approval from both the European Medicine Agency (EMA) and FDA for diagnostic clinical practice [12,20].

In recent decades, nuclear medicine has embraced innovative approaches and methods, particularly with the combined use of diagnostic and therapeutic radiopharmaceuticals, leading to the development of theranostics. Utilizing the same radionuclide or pairs of chemically identical radioisotopes with similar half-lives and complementary emissions has proven to be an efficient strategy for theranostic studies. These shared features ensure the same behavior for both diagnostic and therapeutic radiotracers, a crucial factor contributing to the success of the analysis [21,22,23,24]. An illustrative example is the utilization of [^68^Ga]Ga-DOTA-TOC, which extends beyond diagnostic applications and is also investigated in theranostic use, in conjunction with its therapeutic partner [^177^Lu]Lu-DOTA-TATE (Lutathera^®^), known for its significant binding affinity for somatostatin receptors (SSTR) [12].

In addition to the DOTA chelator [25], various others, such as 1,4,7-triazacyclononane-N,N′,N″-triacetic acid (NOTA) and 1,4,7-triazacyclononane,1-glutaric acid-4,7-acetic acid (NODAGA), have been explored with ^68^Ga for tumor investigations [26].

NODAGA was selected as the bifunctional chelator in our study. NODAGA proves to be optimal for ensuring the stability of ^68^Ga in vivo and is effective as the linker with Nastorazepide (Z-360). Z-360 is well-known for its role as a selective antagonist towards the cholecystokinin b receptor (CCK2R), which serves as the molecular target. In fact, the CCK2R is a G-protein-coupled receptor that is physiologically expressed in some healthy tissues, such as the central nervous system and gastric mucosa. However, it is overexpressed in many cancer tissues, including endocrine, colon and brain tumors, and stromal ovarian and gastrointestinal stromal tumors (GIST). CCK2R plays crucial roles in cancer proliferation, migration and metastasis [27].

Our preclinical study aims to analyze the biodistribution of a newly developed ^68^Ga-labeled radiopharmaceutical, [^68^Ga]Ga-NODAGA-Z360, in an animal model with a human epidermoid carcinoma A431 cell line overexpressing CCK2R (A431 CCK2R+). This analysis involves histopathological and radiomics assessment applied to PET/CT imaging. Histopathological assessments play a crucial role in evaluating the percentage of necrosis in tumor tissues, a significant factor influencing the uptake level and contributing to inhomogeneous or poor uptake [28]. Additionally, histological assessments serve as the gold standard for diagnostic cancer procedure in clinical practice [29,30,31,32]. Conversely, the pathophysiology of targeted organs can be studied with minimal invasiveness through the radiomics approach [33]. The use of radiomics features extracted from images enables the study of the course of tumor diseases or the effects of therapies, providing valuable support for researchers and clinicians in decision-making [25,34,35,36,37]. Our translational approach is reproducible and more reliable, as it replicates the clinical method by integrating histopathology of biopsy samples with radiomics analysis applied to PET/CT images.

**Figure 1:**
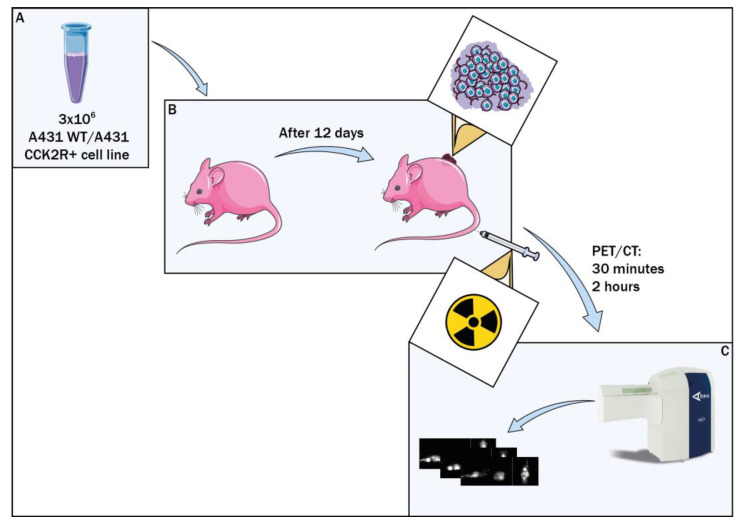
Preclinical study workflow. Balb/c nude mice were xenografted with A431 CCK2R+ cell line, while the control group received A431 WT cells (**A**). Radiopharmaceutical assessment was conducted 12 days post graft (**B**), followed by PET/CT evaluations at 30 min and 2 h post injection (**C**).

We implemented a preclinical radiomics workflow for biodistribution assessment using a specific ^68^Ga-labeled radiopharmaceutical based on [25]. The goal is to enhance the independence of this process from the operator and minimize susceptibility to inter-operator variability in the future. Therefore, conducting numerous experiments using different radiopharmaceuticals and protocols in the field is essential for achieving this goal. To this end, we utilized Balb/c nude mice, a mouse model previously employed in radiopharmaceutical research in oncology [24,25]. In this study, animals were xenografted subcutaneously with human epidermoid carcinoma A431 cells (A431 WT) and with A431 cells overexpressing CCK2R (A431 CCK2R+), cell lines already used for CCK2R-related studies [27,36,38]. To evaluate the [^68^Ga]Ga-NODAGA-Z360 radiopharmaceutical biodistribution, animals underwent PET/CT analyses. A standardized workflow based on our previous studies was developed [37], aiming to make it as translational and reproducible as possible for future clinical trials [39]. This preclinical workflow (Figure 1) utilizes radiomic analyses of PET/CT images of mice to determine the biodistribution of radiopharmaceuticals, offering potential utility for other research endeavors employing various radiopharmaceuticals. This approach reduces the need for invasive procedures, facilitating effective comparisons between preclinical and clinical imaging. In the common clinical practice, the operator is actively involved in the manual segmentation of target organs or tumors. However, our workflow is composed of automatic or semi-automatic steps, which yields to many advantages, such as repeatability of results and enhanced precision in the feature extraction process [17,40,41,42,43,44]. The proposed method assumes a pivotal role in improving traditional analyses, aiming to reduce errors and bridge gaps arising from the diverse scientific backgrounds of researchers who may not always belong to the same sector. By standardizing and automating aspects of the workflow, this method contributes to greater consistency and reliability, ensuring that outcomes are less influenced by individual operator expertise across diverse scientific domains.

## 2. Materials and Methods

### 2.1. Ethics Statement and Animal Model

The experiments followed guidelines set by the European Communities Council directive and Italian regulations (EEC Council 2010/63/EU and Italian D.Lgs. 26/2014). Approval for the project was obtained from the Italian Ministry of Health (authorization number n. No. 44/2021-PR).

We made efforts to minimize the use of laboratory animals by employing replacement, reduction, and refinement measures. Euthanasia was conducted promptly upon reaching the predetermined endpoint to prevent unnecessary suffering in treated mice. This endpoint was determined when tumor lesions exceeded 1.2 cm or when weight loss exceeded 20%. All reasonable measures were taken to alleviate suffering and avoid distressing procedures. To enhance the well-being of the mice and minimize distress, standard environmental enrichment was provided, including two nestles, a card-board Fun Tunnel, and one wooden chew block. The experiments were carried out on 8-week-old Balb/c nude female mice (Charles River Laboratory), weighing 24 ± 3 g. The mice were housed in individually ventilated cage (IVC) systems at a constant temperature (23–25 °C) with a 12/12 h light/dark cycle, and they had ad libitum access to food and water. We maintained a stocking density of 3 mice per cage in individual IVC cages.

The 16 Balb/c nude mice were divided into two groups. Heterotopic tumors were induced in the two groups by subcutaneous injection of 3 × 10^6^ human epidermoid carcinoma A431 cell line (A431 WT) and overexpressing CCK2R (A431 CCK2R+), respectively. The cells were cultured at 37 °C in a humidified environment with 5% CO_2_. Dulbecco modified Eagle medium (DMEM), supplemented with 10% heat-inactivated fetal bovine serum and containing 4.5 g/L glucose, was used for cell growth. The A431 CCK2R+ cells were generated according to a protocol of Aloj et al. [45], and the CCK2R+ cell selection was maintained through the addition of neomycin analog G418 (500 g/mL) to the growth medium. The implantation was carried out using 1:1 (*v*/*v*) of Matrigel and suspended in 100 uL of phosphate buffered saline (PBS).

Mice health was monitored twice a week.

After 12 days post graft (d.p.g.), the radiopharmaceutical was injected into the mice, and the mice underwent μPET/CT scans through Albira Si μPET/CT from Bruker to perform in vivo biodistribution of [^68^Ga]Ga-NODAGA-Z360. Each group was divided into two subgroups, referring to two different PET/CT time points: 30 min or 2 h post injection (p.i.). The selection of the two time points considered the half-life of ^68^Ga radionuclide (68 min) to evaluate the potential changes in radiopharmaceutical biodistribution over time, taking into account the decrease in radiopharmaceutical activity. The μPET/CT procedures were carried out under general anesthesia (isoflurane and oxygen mixture).

After the procedure, mice were sacrificed by cervical dislocation and tissue activity was evaluated through γ-counter. Tumors were also weighed and preserved in paraformaldehyde (PFA), to perform ex vivo tissue analyses.

### 2.2. Immunohistochemistry Staining and Digital Pathology Evaluation

Organs and tumors were collected from euthanized mice, then preserved in 4% paraformaldehyde (PFA), and prepared for embedding in paraffin using Surgipath Paraplast Plus from Leica. These samples were then sectioned for histopathological analysis. The paraffin sections were stained through Hematoxylin–Eosin (H&E; Sigma Aldrich, St. Louis, MI, USA) for overall histopathological evaluations. Whole slide images (WSI) were captured at 20× magnification using the Ventana DP 200 system. The images were stored in RGB color format. Digital pathology necrosis assessments were performed using QuPath v. 0.5.0 software. Manual segmentation of the WSI was assisted by the wand tool of QuPath. For each WSI, tumor necrosis and total tumor regions were segmented, and the area measure was provided by QuPath, shown in μm or pixels. For each sample, percentage (%) of tumor necrosis was measured through the formula (A_n_/A_t_) × 100, in which A_n_ stands for the area of necrosis, while A_t_ represents the total tumor area. Only the tumors with a percentage of necrosis < 50% are included in the following radiomics studies.

### 2.3. Radiopharmaceutical

**Figure 2:**
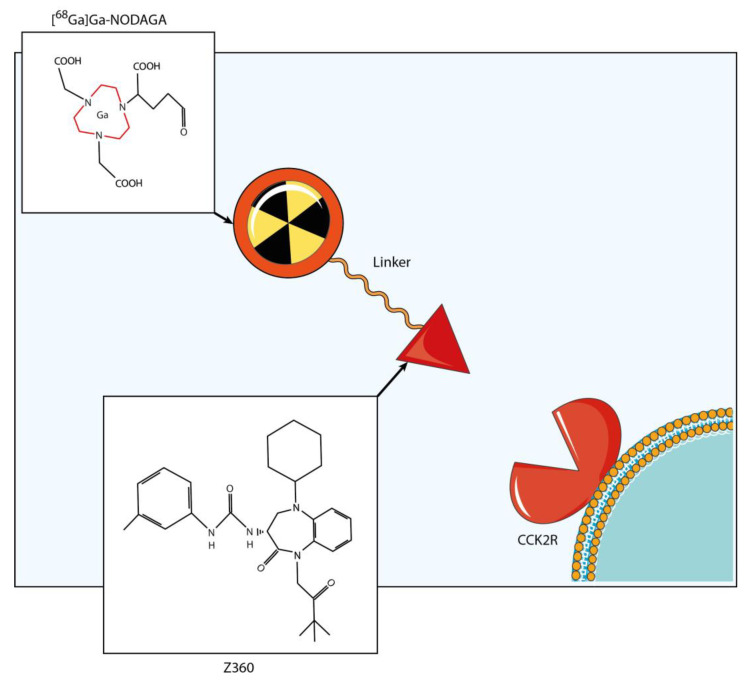
Radiopharmaceutical structure. The radiopharmaceutical consisted of a radionuclide (^68^Ga), a bifunctional chelator (NODAGA) and a targeting vector, known as Z-360. Z-360 is an antagonist of CCK2R, which serves as the molecular target in the study. The labeling reaction was performed at 90 °C, maintaining a pH of 4.5 and achieving a high yield within a 5 min time span.

The radiopharmaceutical structure is composed of a bifunctional chelator with the function to link the ^68^Ga radionuclide to the linker and the directional ligand, represented by the Z360, which is a widely used antagonist of the CCK2R, with the role of driving all the molecule to the CCK2R overexpression site (Figure 2) [44]. Drug labeling with ^68^Ga was carried out at the Cannizzaro Hospital in Catania. To obtain a high yield in a relatively quick time (5 min), the labeling protocol was set at a pH equal to 4.5 and a temperature equal to 90 °C (Figure 2).

### 2.4. PET/CT

Micro-PET/CT (μPET/CT) allows the acquisition of images to study the pathophysiological conditions of small animal models. The dataset was acquired through the μPET/CT (Albira Si Bruker, Ettlingen, Germany), located at the Center for Advanced Preclinical Research (CAPiR), University of Catania, Italy. This preclinical platform allows investigating the pathophysiological conditions of small animal models. The platform was provided by a fully integrated anesthesia system. CT images were acquired at low resolution, through 600 views and 45 mm of initial horizontal position, FOV of 64 mm 35 kV of X-ray energy, and 200 A of current. PET and CT-voxel dimensions were equal to 500 × 500 × 500 mm^3^. The images were saved in Digital Image Communications in Medicine (DICOM) and then reconstructed by 3D-MLEM algorithm, with 12 iterations.

### 2.5. Atlas-Based Multi-Organ Segmentation

**Figure 3:**
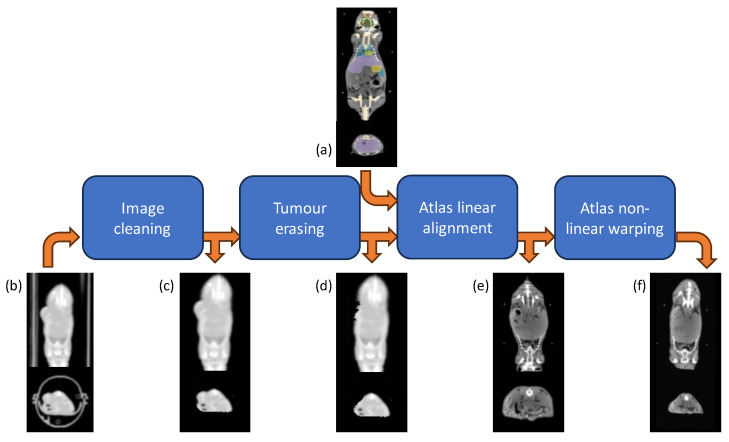
Atlas-based segmentation workflow. Each original CT scan (**b**) underwent first pre-processing for animal holder removal (**c**) and tumor erasing (**d**); then the multi-organ segmentation was accomplished by warping the Digimouse CT atlas (**a**) through an affine transformation (**e**), followed by a B-spline and thin-plate spline mappings (**f**).

The segmentation process was conducted for all co-registered PET/CT scans by employing co-registration techniques with a standard template space, as illustrated in Figure 3, to extract radiomics features from each organ of interest or tumor and to carry out a non-invasive biodistribution analysis based on PET images.

Initially, spatial preprocessing of the CT images was performed using a proprietary MATLAB^®^ [46] algorithm. This preprocessing aimed to remove non-mouse-related structures from the CT scans, including the animal holder, while adjusting the CT image intensity range from the Hounsfield scale to an 8-bit grayscale format. Subsequently, manual contouring and removal of subcutaneous tumor masses from the CT images were performed to mitigate potential shape-related confounding effects during the atlas warping process. In specific cases where the growth of the tumor, implanted subcutaneously, causes inward pressure, the exclusion of these tumor areas allowed the atlas to better account for these variations in shape.

Following the preprocessing steps, a three-step registration procedure aligned the 3D whole-body Digimouse atlas [47] with each CT scan. This template was deemed suitable due to its alignment with the anatomical mouse model and its construction from similar imaging modalities (PET, X-ray CT, and cryosection images of normal nude mice). However, in our co-registration approach, only the CT component of the atlas was utilized, as its functional part relates to the PET uptake, thereby potentially influencing biodistribution patterns and subsequently affecting study results.

The segmentation process itself was conducted in the native subject space rather than the template space [37,48]. This approach involved registering the atlas to each CT image and propagating the labels accordingly. This method was preferred over image normalization to the atlas space due to the low resolution of the CT scans and to circumvent PET warping and interpolation, which could adversely impact subsequent feature extraction stages. The registration process encompassed a semi-automated linear alignment utilizing ITK-Snap software (www.itksnap.org) [49], followed by an automated non-linear warping using the Elastix toolkit [50]. Additionally, small local refinements were performed through visual inspection and achieved using the landmark registration tool of 3DSlicer [51]. To elaborate further, each mouse’s atlas underwent manual pre-alignment to correspond to the subject-specific coordinate system through rigid roto-translation. This pre-alignment aimed to facilitate the subsequent automated algorithm convergence. Subsequently, an optimal affine transform, employing mutual information as the similarity metric with a multi-resolution approach (utilizing half-resolution at the coarsest level and full resolution at the finest, due to raw data quality limitations), was estimated. Following this, a non-linear intensity-based registration using a B-spline deformation model was conducted based on metric, optimization routine, and parameter settings, as detailed in [52]. This comprehensive procedure notably enhanced the alignment of major anatomical structures with high contrast, such as the spine, skull, and limbs. However, anticipated misalignments in low-contrast tissues of interest, such as the bladder, and minor residual differences in lung contour necessitated local refinements. Thus, a thin-plate spline mapping, achieved through multiple landmark definitions positioned manually by visual inspection, was generated.

Finally, the linear and non-linear transformations estimated throughout these processes were utilized to warp the binary masks of the selected volume of interest (VOIs) into each subject-specific space.

### 2.6. Radiomics Feature Extraction and Analyses

Following the co-registration process outlined previously, the organs of interest (such as the heart, bladder, stomach, spleen, liver, kidneys, and lungs) along with tumors were identified and saved as binary masks, with the background labeled as 0 and the organ of interest labeled as 1. Before extracting features, PET DICOMs underwent modifications to include standardized uptake value (SUV), as detailed in references [53]. SUV is a widely used semi-quantitative parameter for estimating biodistribution in PET images. It normalizes voxel activity by considering acquisition time, administered activity, and mouse weight. Essentially, PET images were converted into SUV images, allowing for the incorporation of factors that would otherwise be overlooked during radiomics analysis. Using PET and co-registered masks, a total of 112 radiomics features were extracted utilizing an image biomarker standardization initiative (IBSI) [54] -compliant analysis software, i.e., PyRadiomics [55], for increasing the reproducibility of the extracted features. This is a crucial aspect of radiomics studies [53]. PyRadiomics is a Python-based open-source program designed for scientific computing, compatible with various platforms. The software extracted various types of features, including shape descriptors, first-order statistics, and texture matrices such as gray-level co-occurrence matrix (GLCM), gray-level run-length matrix (GLRLM), gray-level dependence matrix (GLDM), gray-level size-zone matrix (GLSZM), and neighboring gray-level dependence matrix (NGLDM). Shape descriptors are concerned with the geometric characteristics of the objects in the image and are not influenced by the intensity distribution of gray levels. These descriptors encompass attributes such as volume, maximum diameter, surface area, compactness, and sphericity. First-order statistical descriptors, also known as histogram-based features, analyze the frequency distribution of voxel intensities within an organ by examining the histogram of gray-level intensity values.

Texture features, on the other hand, provide insights into the spatial arrangement of gray levels within the image. They evaluate the relative positions of voxels, offering information about the spatial organization of gray levels within the organ of interest.

The performance of the proposed approach was evaluated using the unpaired *t*-test. This statistical test is employed to determine whether there is a significant difference between the means of two independent groups.

In other words, the unpaired *t*-test compares for each group (in our case, the four different conditions at 2 h, at 30 min, regardless of the time points, and regardless of the WT and CCK2R groups), in each individual organ/tumor, the means of each of the 112 features, determining whether one of these means represents a statistically significant variation compared to the others. Finally, radiomics features with a p-value of less than 0.05 were deemed significant. These features were examined to determine the percentage of variation among the 112 overall features for each individual organ. This process aimed to identify which features exhibited statistically significant changes across the four pairs of groups under consideration.

## 3. Results and Discussion

**Figure 4:**
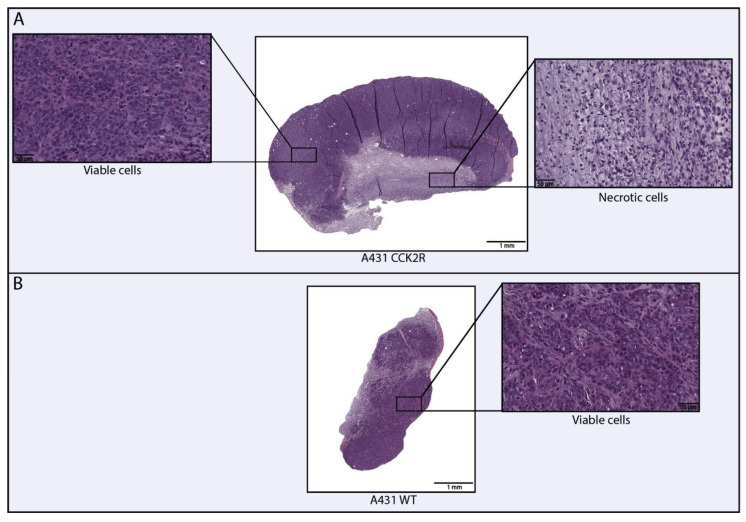
Digital evaluation of necrosis in human epidermoid carcinoma A431 WT and A431 CCK2R tumors. The figure illustrates the necrotic and viable areas within the tumors. A431 CCK2R+ tumors exhibit approximately 15–20% of necrosis relative to the total area (**A**), whereas A431 WT tumors show an absence of necrosis (**B**).

The Balb/c nude mouse model was xenografted with human epidermoid carcinoma cell lines (A431) WT and overexpressing CCK2R, the molecular target of the study. Following euthanasia, organs and tumors were harvested and processed for histopathological H&E staining. WSI evaluation, performed through digital pathology, revealed the rapid growth rate of the A431 CCK2R+ cell line, resulting in extensive necrosis within the tumor core (Figure 4). A necrotic tumor is characterized by rapid growth that exceeds the capacity for angiogenesis, resulting in the inability to vascularize the entire tumor area [28]. There is an impact of necrosis on the uptake of radiopharmaceuticals, even in CCK2R tumors, which hampers the targeting of radiopharmaceuticals and contributes to radio-resistance [28,56]. Segmentation using QuPath allows for the calculation of living tumor areas in WSIs compared to the total tumor areas and their respective percentages. Tumors with high necrosis areas (>50%) were excluded from the radiomics analysis, focusing only on tumors with minimal necrosis areas. The remarkably high level of necrosis in these tumors may distort radiopharmaceutical uptake, resulting in an inhomogeneous and potentially misleading signal [28].

This specific analysis provided information on the absence of necrosis in WT as opposed to CCK2R tumors, which exhibited a necrotic area ranging from 15–20%.

The investigated radiopharmaceutical consisted of the ^68^Ga radionuclide labeling a Z360 targeting vector, [^68^Ga]Ga-NODAGA-Z360. Z360 is a widely used antagonist of the CCK2R to drive the whole molecule to the CCK2R overexpression sites. For a minimally invasive assessment of radiopharmaceutical biodistribution based on PET/CT images of animal models, the previous established workflow, tested with ^64^Cu, was employed [25]. Moreover, high co-registration accuracy was achieved, including intensity correlation between the warped atlas and the CT images [37].

**Figure 5:**
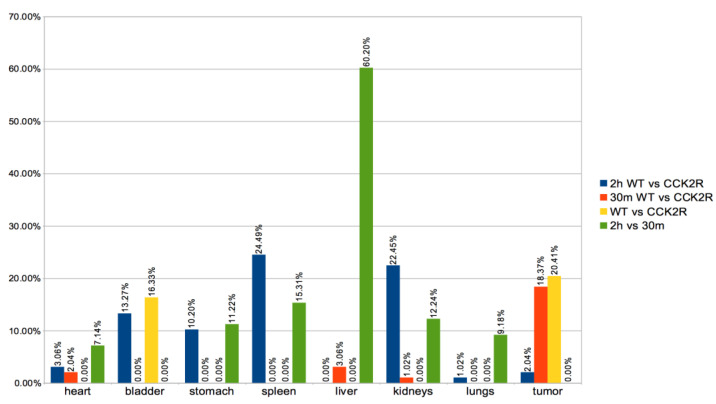
Differences in radiomics features between groups. A variety of organs were considered for the extraction of radiomics features, such as the heart, stomach, liver, spleen, lungs, kidneys, bladder, and tumors. Radiomics analysis of 112 features of PET sequence expressed as % of variation between the WT and CCK2R groups for each organ/tumor were performed at both 2 h (blue) and 30 min (red). In addition, the feature variation in % between WT and CCK2R mice for each organ/tumor was analyzed independently of the time points (yellow), and the feature variation in % between 2 h and 30 min for each organ/tumor was analyzed independently of the WT and CCK2R groups (green). Differences between groups are considered significant for *p* < 0.05 (unpaired *t*-test).

In this study, PET/CT images and radiomics analyses were used to evaluate biodistribution of the radiopharmaceutical 30 min and 2 h after injection. The radiomics analysis allowed us to identify 112 radiomics features in each different body district. Radiomics variation in percentage were evaluated in the tumor and each organ, including heart, stomach, liver, spleen, lungs, kidneys, and bladder (Figure 5). For a more consistent interpretation of the biodistribution results, the analyses were performed both dependently and independently of the time point. Notably, considering WT and CCK2R tumors, a statistically significant (*p*-value < 0.05) high variation in features percentage was detected in bladder, stomach, spleen, and kidneys at 2 h (see blue bar in Figure 5); instead, a statistically significant high variation in features percentage was detected in tumors at 30 min (see red bar in Figure 5).

Conversely, in a completely time-independent manner (see yellow bar in Figure 5), a distinct trend was observed in tumors and bladder, with more prominent statistically significant (*p*-value < 0.05) variation in radiomics features between the WT and CCK2R groups. The observed trend is probably related to the radiopharmaceutical biodistribution. Specifically, in CCK2R tumors, an early accumulation was hypothesized, leading to subsequent movement of the radiopharmaceutical toward the excretion pathway through the bladder.

Moreover, it is worth noting that a statistically significant variation of feature percentage was detected in bladder, stomach, spleen, and kidneys at 2 h versus to 30 min (see green bar in Figure 5).

**Figure 6:**
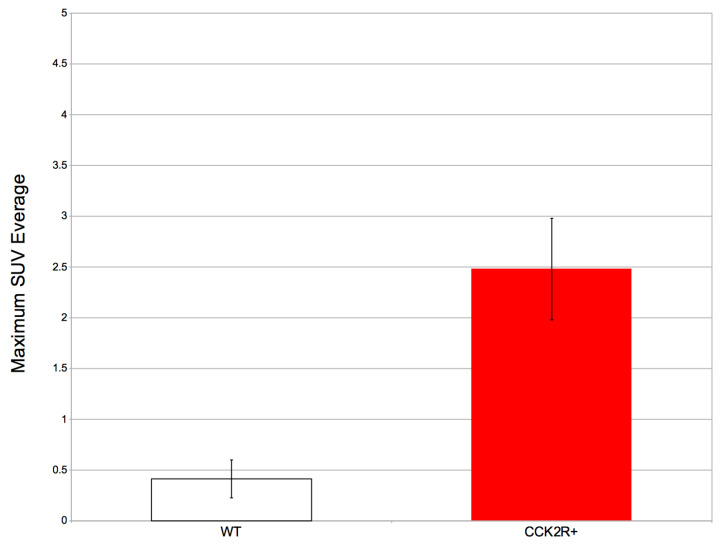
The average SUVmax and standard deviation in WT and CCK2R+ tumors. Differences between tumors are considered significant for *p* < 0.05 (unpaired *t*-test).

These findings are corroborated through a focused analysis, particularly emphasizing a specific radiomics feature: the SUV. SUV is the most used quantitative measure used in PET imaging to assess the concentration of a radiopharmaceutical within a particular tissue or lesion. It is calculated by normalizing the radioactivity concentration in the VOI to the injected dose of the radiopharmaceutical and the patient’s body weight [57]. SUVmax and SUVmean are the most commonly used SUVs [58]. Specifically, SUVmax, being referred to only one voxel of the tissue or lesion, is considered more reproducible than SUVmean and therefore is more commonly used as a PET parameter [59,60]. In our study, the SUVmax assessment revealed that the CCK2R+ tumors had a significant radiopharmaceutical uptake compared to WT tumors, which do not present uptake in any of the mice considered with a *p*-value < 0.05 (Figure 6). These data results are very promising, since they confirm the biodistribution of the [^68^Ga]Ga-NODAGA-Z360 radiopharmaceutical towards the site of CCK2R overexpression, as expected.

**Figure 7:**
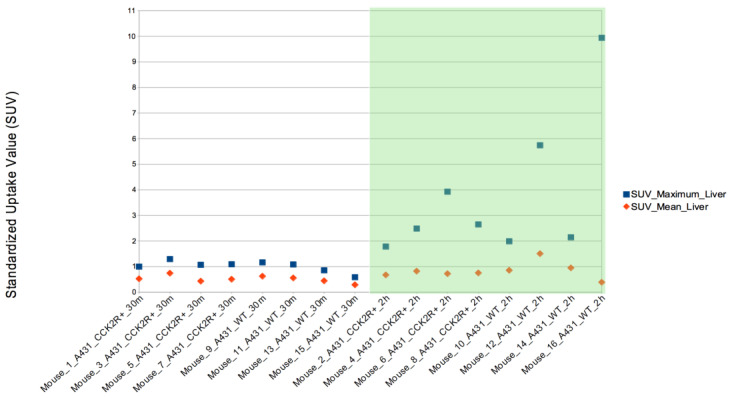
SUVmax evaluation in liver. The SUVmax trend was significantly higher at 2 h than at 30 min post injection for both CCK2R and WT. Differences are considered significant for *p* < 0.05 (unpaired *t*-test).

**Figure 8:**
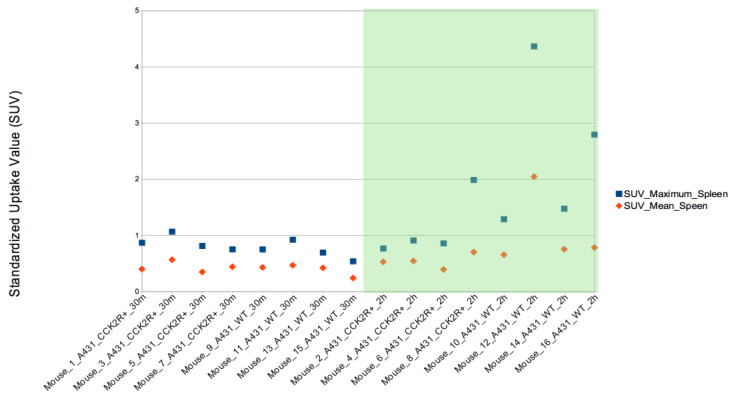
SUVmax evaluation in spleen. The SUVmax trend was significantly higher at 2 h than at 30 min post injection for both CCK2R and WT. Differences are considered significant for *p* < 0.05 (unpaired *t*-test).

In addition, SUVmax was evaluated in the other body districts, including heart, stomach, liver, spleen, lungs, kidneys, and bladder. Among these, SUVmax exhibited significant variation between the 2 h and 30 min time points in both the liver and spleen, illustrating a progressive increase in uptake over time (Figure 7 and Figure 8) since the radiotracer had more time to accumulate in the organs under observation. The difference between SUVmean and SUVmax might increase over time, because SUVmean considers all the voxels within the target, including those with lower radiopharmaceutical uptake, while SUVmax is based only on the voxel with the highest uptake intensity.

## 4. Conclusions

Advancements in the oncological field have relied on the integration of diverse multidisciplinary technologies. Oncology researchers are exploring various methods to overcome the technologies of current medical practice by looking for alternatives to conventional approaches [60]. In the last years, nuclear medicine has been considerably expanding, offering significant advancements in precision medicine methods. Our study aligns with these premises, introducing unbiased methods of analysis through the application of radiomics and digital pathology in preclinical studies and investigating the in vivo biodistribution of a ^68^Ga-labeled radiopharmaceutical, [^68^Ga]Ga-NODAGA-Z360, through μPET/CT imaging. The Balb/c nude mouse model was employed to ensure the xenograft with human cell lines (A431) WT and overexpressing CCK2R, the target molecule of the study. The mice were analyzed through μPET/CT at two time-points (30 min and 2 h), directly related to the short radionuclide half-life. As a result of applying our minimally invasive workflow to this new study on targeted radiotherapy with a ^68^Ga-labeled radiopharmaceutical, the evaluation of biodistribution through radiomics appears to be exhaustively validated in the preclinical setting. In conclusion, the promising results of this study took into account certain limitations, primarily derived from biological issues, which could be solved in future studies. Specifically, our study focused on evaluating the biodistribution in a single mouse model, Balb/c nude xenografted with the human epidermoid carcinoma A431 cell line, commonly used in preclinical radiopharmaceutical studies [27,28]. However, the study may be enhanced by assessing the radiopharmaceutical biodistribution through different models, including patient-derived xenograft (PDX) models or orthotopic genetically-engineered mouse models (GEMM) of CCK2R+ gastrointestinal tumors, which are considered more accurate and closely mimic the human cancer counterparts [61,62]. Moreover, the inclusion of additional immunohistochemical methodologies, such as microvessel density assessment, could provide valuable insights. Despite biological limitations, which must always be considered when evaluating experimental results, our analysis method demonstrated promising results for translational applications. In the future, it would be interesting to conduct further research to study the biodistribution of radiopharmaceuticals under different conditions and with different time curves of drug administration. In contrast to traditional methods, radiomics feature extraction considers a greater number of parameters. This allows us to obtain more information not only about radiopharmaceutical uptake but also about other factors reflecting tumor characteristics. The radiomics approach is already used for predicting and comparing patients in the clinical setting, so the results obtained in animal models are even more translational because radiomics can perform quantitative assessments of the images, as well as detect characteristics not normally visible to the naked eye by the expert researcher/anatomical pathologist/radiologist/veterinarian. Finally, further investigations into the biological pharmacokinetics of the radiopharmaceutical will be carried out in a future study to provide a comprehensive understanding of its biological dynamics.

## Data Availability

Data are contained within the article.
